# Micro RNA-640 Targeting SLIT1 Enhances Glioma Radiosensitivity by Restraining the Activation of Wnt/β-Catenin Signaling Pathway

**DOI:** 10.3389/bjbs.2022.10067

**Published:** 2022-04-07

**Authors:** Yamei Zheng, Mingyue Xiao, Jingqiong Zhang, Fei Chang

**Affiliations:** ^1^ Department of Oncology, The Central Hospital of Wuhan, Tongji Medical College, Huazhong University of Science and Technology, Wuhan, Hubei, China; ^2^ Department of Neurology, The Central Hospital of Wuhan, Tongji Medical College, Huazhong University of Science and Technology, Wuhan, Hubei, China

**Keywords:** MiR-640, SLIT1, radiosensitivity, glioma, wnt/β-catenin signaling pathway

## Abstract

**Purpose:** The purpose of this study was to analyze the effects of miR-640–SLIT1 axis and the Wnt/β-catenin signaling pathway on radiosensitivity of glioma cells.

**Methods:** Relative expressions of miR-640 and slit guidance ligand 1 (SLIT1) in glioma tissues and glioma cell lines U251 and A172 were detected using RT-qPCR. The cell lines were transfected with si-SLIT1 or miR-640 inhibitor to study the radiosensitivity of glioma cells. We detected cell activity using CCK-8 assay, cell migration using wound healing assay, cell invasion using transwell assay, and apoptosis using caspase-3 assay.

**Results:** SLIT1 was upregulated in glioma tissues and cell lines, and inversely correlated with radiation sensitivity. Its knockdown reduced radioresistance, migration, and invasion, but increased apoptosis in U251 and A17 cells. Loss of miR-640 activity upregulated SLIT1, Wnt, and β-catenin protein expression, whereas it inhibited p-GSK-3β protein levels in U251 and A17 cells. These results suggest that miR-640 mediates the radiosensitivity of glioma cells through SLIT1 and the Wnt/β-catenin signaling pathway.

**Conclusion:** The miR-640–SLIT1 axis that regulates the Wnt/β-catenin signaling pathway is a possible therapeutic option for the effective treatment of glioma in combination with radiotherapy.

## Background

Glioma accounts for more than 50% of brain tumors and is the most severe central nervous system disorder affecting adults (1). At present, its annual incidence is approximately 5 cases per 100,000 people, making it one of the deadliest tumors globally (2, 3). Radiation sensitivity is often a decisive factor for prognosis in glioma patients (4, 5). In many instances, the outcome of radiotherapy in these patients is only partially effective, which may be due to inherent or acquired radio-resistance (6). Therefore, enhancing the radiosensitivity of glioma cells and finding effective therapeutic targets is of paramount importance.

Micro RNAs (miRNAs or miRs) contribute to whole-body homeostasis by regulating cell growth, apoptosis, metabolism, tumorigenesis, and cell cycle progression (7). They promote or restrict cancer development. For example, miR-100-5p inhibits proliferation, migration, and invasion of prostate cancer cells (8). MiR-1256 is downregulated in papillary thyroid cancer, and restoring its expression inhibits cancer cell growth by repressing proliferation-associated proteins including PCNA, CDK4, and cyclin D1 (9). Evidence suggests that several miRNAs also have anti-radiation resistance effects in tumors. For instance, miR-16-5p, a candidate tumor suppressor, or miR-153-3p enhances the sensitivity of glioma cells to irradiation (10, 11). Of the known human miRNAs involved in cancer, miR-640 is the least studied. Its expression is low in hepatocellular carcinoma (HCC) tissues, which contributes to the malignant behavior of HCC cells (12). Because it promotes inflammation, its effect on the inflammatory response is currently the focus of extensive research (13, 14). However, whether miR-640 is implicated in radio-resistance of glioma is still unknown.

The highly conserved secreted glycoproteins slit guidance ligand (SLIT) play a vital role in many biological processes, including angiogenesis and cell migration (15). Tri-methylation of SLIT1 promoter inhibits the proliferation and metastasis of colon cancer cells (16). This tumor-suppressive feature of the SLIT1 gene has also been observed in gliomas (17). The effect of SLIT1 on cancer radiosensitivity has not been reported yet.

In the complex molecular response of cancer cells to radiation, activation of the Wnt/β-catenin pathway induces DNA damage repair and inhibits cell apoptosis, conferring tumor radiation resistance (18, 19). In the radiation-resistant esophageal cancer cells, epithelial-mesenchymal transition is promoted by activating the Wnt/β-catenin signaling pathway, suggesting that the activation promotes radiation resistance (20). Conversely, inactivating the Wnt/β-catenin signaling pathway inhibits the proliferation of non-small cell lung cancer cells to induce radio-sensitization (21). In addition, the Wnt/β-catenin signaling pathway is usually activated in glioma (22). Therefore, we investigated the effects of miR-640 and SLIT1 on the Wnt/β-catenin signaling pathway and radiation-triggered cellular responses as a possible mechanism of miR-640 induced radio-sensitization.

## Materials and Methods

### Tissue Samples

In this study, 34 patients with gliomas who underwent surgery in our hospital from January 2019 to January 2020 were enrolled. The tumor tissue and the adjacent normal brain tissue 2 cm away from the glioma were collected from all patients. At least two pathologists diagnosed the glioma tissue and identified the normal tissue. Each excised tissue sample was rapidly frozen in a test tube in liquid nitrogen and stored at −80°C until RNA extraction. The 34 gliomas were classified according to the 2016 WHO classification (23) as follows: grade I: 4 pilocytic astrocytomas; grade II: 1 diffuse astrocytoma, 2 oligoastrocytomas, and 3 oligodendrogliomas; grade III: 4 anaplasia astrocytomas, 7 oligoastrocytomas, and 2 oligodendrogliomas; and grade IV: 8 primary glioblastomas and 3 secondary glioblastomas. All patients received radiotherapy before surgery, and none had a prior history of brain tumors. The patients gave informed consent before sample collection, and the ethics committee of The Central Hospital of Wuhan approved all the experiments (approval number: TJ-IRB201909113). The clinicopathological features of the patients are shown in Supplementary Table S1.

### Cell Culture

Human glioma cells, U251 and A172, and astrocyte cell line NHA were obtained from the American Type Culture Collection (VA, United States). The cells were cultured in DMEM (Gibco, CA, United States) medium containing 10% FBS and 1% penicillin–1% streptomycin (Gibco). They were maintained in an incubator with 5% CO_2_ at 37°C.

### Cell Transfection

U251 and A172 cells were transfected using Lipofectamine 2000 (Invitrogen, CA, United States) with 100 nM miR-640 inhibitor or its negative control (inhibitor-NC) or 75 nM of a plasmid expressing siRNA against SLIT1 (si-SLIT1) or its negative control (si-NC). Genewiz Biotechnology Co., Ltd. (Suzhou, China) synthesized the inhibitor, plasmid, and controls.

### Irradiation Treatment

U251 and A172 cells were cultured in a 96-well plate at a 1 × 10^4^/well density. The cells were placed in a linear accelerator (Rad Source, GA, United States) and irradiated with a 6 MV photon beam at a 3.2 Gy/min dose rate. The X-ray radiation dose was 2, 4, 6, or 8 Gy. After each irradiation, the cells were cleared with PBS and cultured on alternative media, followed by RT-qPCR analysis within the next 24 h.

### Real-Time Quantitative PCR

Micro RNA was extracted by NucleoSpin miRNA kit (Macherey–Nagel, France) and total RNA by an RNA isolation kit (Takara Bio, Japan). A260/A280 absorbance ratio was quantified to assess RNA purity. Micro RNA was reverse-transcribed into cDNA by the ImProm-II Reverse Transcription System (Promega, WI, United States). TransStart Eco Green qPCR SuperMix (TransGen Biotech, Beijing, China) was used in a CFX Connect Real-Time PCR Detection System (Bio-Rad, United States) for PCR reaction. PrimeScript RT Master Mix (Takara Bio) was used to reverse transcribe mRNA into cDNA. The relative expression of miRNA and mRNA was quantified by the 2^−∆∆CT^ method (24) and normalized to endogenous reference gene U6 and GAPDH, respectively. Primer sequences for this study are shown in Table 1.

**TABLE 1:** Primer sequences for this study.

Gene	Primer sequences	
SLIT1	forward	5′-GAC​GTG​GTC​TGT​CCC​CAC​AA-3′
	reverse	5′-AAT​CTC​ATT​GTT​ATT​CAA​TCG​CAG​TT-3′
miR-640	forward	5′-GGA​TGA​CCA​TGA​CCT​TTG​GT-3′
	reverse	5′-ACC​TAA​GCC​AGG​GAG​GTT​A-3′
GAPDH	forward	5′-TGC​CCC​CAT​GTT​CGT​CA-3′
	reverse	5′-CTT​GGC​CAG​GGG​TGC​TAA-3′
U6	forward	5′-CTC​GCT​TCG​GCA​GCA​CA-3′
	reverse	5′-AAC​GCT​TCA​CGA​ATT​TGC​GT-3′

### Western Blot Assay

The western blot assay was conducted as previously reported (25). Total protein was isolated from U251 and A172 cell lines with RIPA lysis buffer (Invitrogen) and quantified using the bicinchoninic acid (BCA) method. The proteins were separated by 10% SDS-PAGE. Equal amount of protein was transferred to a PVDF membrane and incubated at 4°C for 24 h with anti-SLIT1 (1:5,000, ab151724, Abcam, United Kingdom), anti-Wnt (1:1,000, ab15251, Abcam), anti-β-catenin (1:5,000, ab19381 Abcam), anti-p-Gsk-3β (1:1,000, ab131097, Abcam), anti-Gsk-3β (1:1,000, ab93926, Abcam), or anti-GAPDH (1:2000, ab8245, Abcam). The membrane was incubated with HRP-conjugated IgG secondary antibody (1:10,000, ab97051, Abcam) at room temperature for 2 h. The protein blots were visualized using the ECL method. GAPDH served as an internal control.

### CCK-8 Assay

The viability of U251 and A172 cells was determined using a CCK-8 kit (Sigma). Briefly, the CCK-8 solution was directly added to the cultured cells after transfection or irradiation to produce a yellow formazan dye. The absorbance at 450 nm was determined by a microplate reader (BioTek Instruments, VT, United States). The amount of formazan dye was directly proportional to the number of viable cells.

### Wound Healing Assay

The treated U251 and A172 cells were seeded into a 6-well plate and cultured until almost complete confluence. A single cell layer was scraped with a 100 μl sterile pipette tip to create the artificial wound surface. The cells were washed with PBS and then cultured in a fresh serum-free medium. Photographs were taken under an optical microscope (Olympus, Tokyo, Japan) at 0 and 24 h, and the wound spacing was measured.

### Cell Invasion Assay

The treated U251 and A172 cells were diluted with a serum-free medium and implanted at a 1 × 10^5^ density into the upper chamber of a Matrigel-precoated transwell insert (Corning, MA, United States). The lower chamber was filled with a 500 μl complete medium containing 10% FBS as a chemical attractant. After culturing for 24 h, the cells on the upper membrane surface were wiped off with cotton, and the cells on the lower fixed with methanol. The fixed cells were stained with 0.1% crystal violet for 2 h. The invading cells were counted and photographed under a microscope.

### Caspase-3 Activity Assay

The activity of caspase-3 was detected using a caspase-3 colorimetric kit (Abcam, MA, United States). Briefly, the treated U251 and A172 cells were trypsinized and centrifuged at 1,800 ×g for 10 min. The cells were washed in cold PBS, dissolved in 50 μl solution buffer, and centrifuged at 10,000 ×g at 4°C for 1 min. After quantifying the total protein by the BCA method, 100 μg of protein was mixed with a 50 μl reaction buffer containing DTT and a caspase-3 substrate (DEVD-pNA) and cultured at 37°C for 12 h. Caspase-3 activity was measured in a cell lysis buffer on a plate reader at 405 nm.

### Luciferase Reporter Assay

The luciferase reporter gene was subcloned into the pLG3 vector (Promega). U251 and A172 cells were seeded on a 6-well plate at a 1 × 10^5^ density. The wild-type pGL3-SLIT1 3′ UTR plasmid (Wt) containing a miR-640 binding site, mutant pGL3-SLIT1 3′ UTR plasmid (MUT1, MUT2, and Co-Mut), mutated via the QuikChange II XL Site-Directed Mutagenesis Kit (Stratagene, Santa Clara, CA, United States), and a miR-640 mimic were co-transfected with Lipofectamine 2000 reagent into U251 and A172 cells. After 48 h transfection, luciferase activity, produced by the different vectors, was detected with the Dual-Luciferase Reporter Assay System (Promega). Relative luciferase activity was expressed as the ratio of firefly to Renilla luciferase activity.

### Biotinylated RNA Pull-Down Assay

Genechem Co., Ltd. (Shanghai, China) synthesized a biotin-labeled miR-640 (Bio-miR-640 mimic) and the negative control (Bio-NC). U251 and A172 cells were transfected with either the biotinylated miR-640 or the negative control for 48 h using the Lipofectamine 2000 reagent. After transfection, the cells were lysed with a lysis buffer, and the cell lysates were incubated with streptavidin-conjugated agarose beads (Invitrogen). The relative expression of the bound SLIT1 mRNA was quantified using RT-qPCR.

### Statistical Analysis

The data were based on at least three independent experiments and were expressed as mean ± standard deviation. Statistical analysis was performed on the SPSS 10.0 software (SPSS, IL, United States). The comparison of miR-640 or SLIT1 in cancer and normal tissues was performed using the paired Student’s t-test. Correlation analysis between miR-640 and SLIT1 in cancer tissues was performed by Pearson correlation. A one-way ANOVA and Tukey’s test were used for the statistical analysis of cells. Differences were considered statistically significant when *p* < 0.05.

## Results

### SLIT1 is Upregulated in Glioma and Associates With Radiation Resistance

**FIGURE 1:**
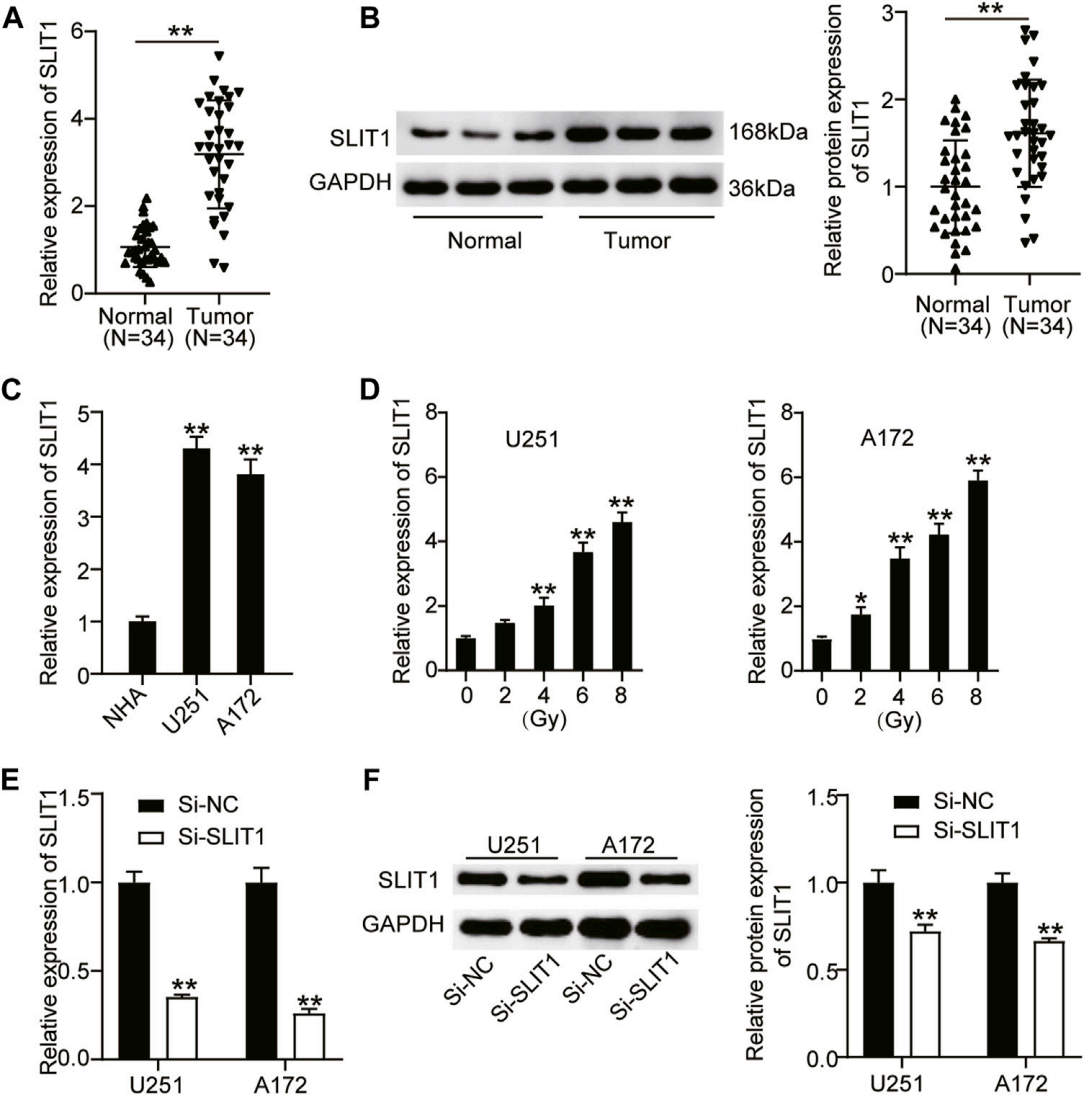
SLIT1 was upregulated in glioma and was associated with radiation resistance. **(A)** Quantitative real-time (qRT)-PCR was carried out to determine the expressions of SLIT1 in glioma tissues and matched adjacent normal tissues. **(B)** Western blotting was carried out to determine the protein expressions of SLIT1 in glioma tissues and matched adjacent normal tissues. **(C)** The expressions of SLIT1 in glioma cells (U251 and A172) and normal human astrocytes NHA cell line were evaluated by qRT-PCR. **(D)** The expressions of SLIT1 in glioma cells were examined by qRT-PCR within 24 h after 0, 2, 4, 6, 8 Gy irradiation. **(E)** The expression of SLIT1 in U251 and A172 cells transfected with Si-SLIT1 was examined by qRT-PCR. **(F)** The protein expression of SLIT1 in U251 and A172 cells transfected with Si-SLIT1 was measured by western blot. *p < 0.05; **p < 0.001.

To define the biological role of SLIT1 in radiation-resistant glioma, we first analyzed its expression in glioma and adjacent normal tissues from the patients who underwent radiotherapy using RT-qPCR and western blotting. Compared with normal tissues, SLIT1 mRNA and protein expression levels in glioma tissues were significantly elevated by approximately 3.5- and 1.8-fold, respectively (Figures 1A,B). Furthermore, we examined *SLIT1* expression in glioma cells and found that it was increased in U251 and A172 cells compared with that in healthy NHA cells (Figure 1C). These results suggest that SLIT1 is upregulated in glioma. Next, we treated U251 and A172 cell lines with different doses of X-rays to explore their effect on SLIT1 expression in glioma cells. Remarkably, increasing the radiation dose enhanced SLIT1 expression in the cell lines, revealing its possible association with radiation resistance (Figure 1D). To elucidate the relationship between SLIT1 and glioma radiation resistance, we knocked down SLIT1 in the U251 and A172 cell lines. By transfecting si-SLIT1 into the cells, we found that the SLIT1 mRNA expression was reduced by more than 60% compared with the mock-transfected cells (Figure 1E). A Western blot assay also showed a decrease in SLIT1 protein levels, confirming SLIT1 silencing in the cells (Figure 1F).

### Low SLIT1 Expression Inhibits Tumorigenicity and Promotes Radiosensitivity of Glioma Cells

**FIGURE 2:**
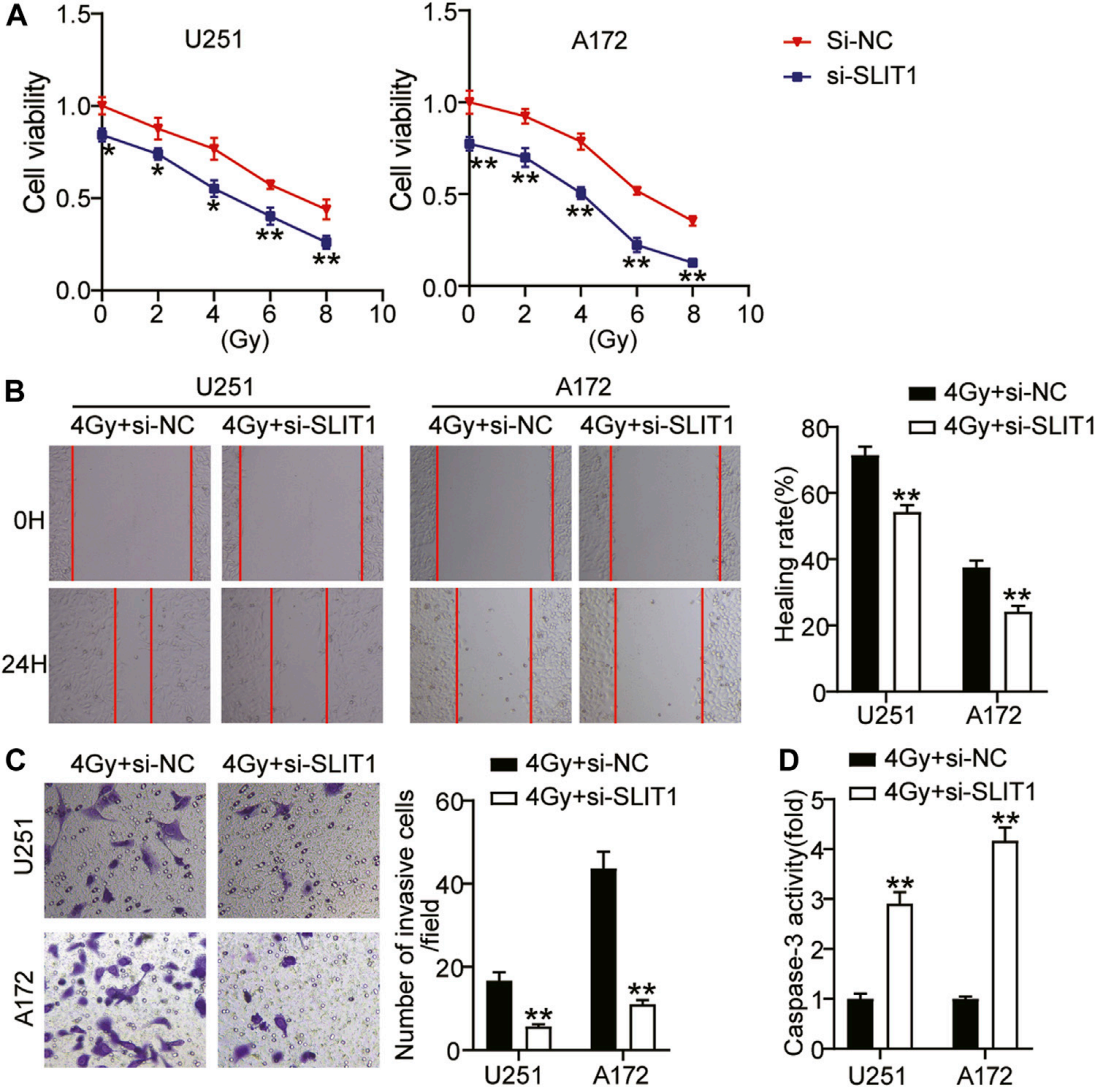
Low expression of SLIT1 inhibited the tumorigenesis and improve radiosensitivity of glioma cells. **(A)** The cell viability curves of U251 and A172 cells transfected with Si-SLIT1 were established by the CCK-8 assay. **(B)** Cell migration rate at 0 and 24 h in U251 and A172 cells transfected with Si-SLIT1 were detected by wound healing assay. **(C)** Transwell invasion assay was performed to determine cell invasiveness of U251 and A172 cells transfected with Si-SLIT1. **(D)** Caspase-3 activity was assessed by caspase-3 activity assay in U251 and A172 cells transfected with Si-SLIT1. *p < 0.05; **p < 0.001.

Aiming at the up-regulation of SLIT1 expression in glioma cells and the change of SLIT1 expression under the action of radiation, we further studied the role of SLIT1 in the malignant behavior and radiosensitivity of glioma cells through CCK-8, wound healing, and transwell assays. CCK-8 assay showed that the viability of U251 and A172 cells decreased with increasing radiation doses. Moreover, SLIT1 knockdown significantly affected cell viability at low and high radiation doses (Figure 2A). We selected 4 Gy as the fixed radiation dose and further studied SLIT1 effects on glioma radiosensitivity. Wound healing assay showed that SLIT1 knockdown inhibited U251 and A172 cell migration by about 30% at 4 Gy irradiation (Figure 2B). In addition, the transwell assay revealed that SLIT1 silencing significantly inhibited the invasiveness of the cells (Figure 2C). To assess the influence of SLIT1 on the apoptosis of glioma cells, we analyzed the activity of caspase-3 in the U251 and A172 cell lines. When SLIT1 was knocked down, the caspase-3 activity of the cells significantly increased under 4 Gy (Figure 2D). These data indicate that low SLIT1 expression inhibits the tumorigenicity of glioma cells and increases their radiosensitivity.

### miR-640 Binds and Negatively Regulates SLIT1

**FIGURE 3:**
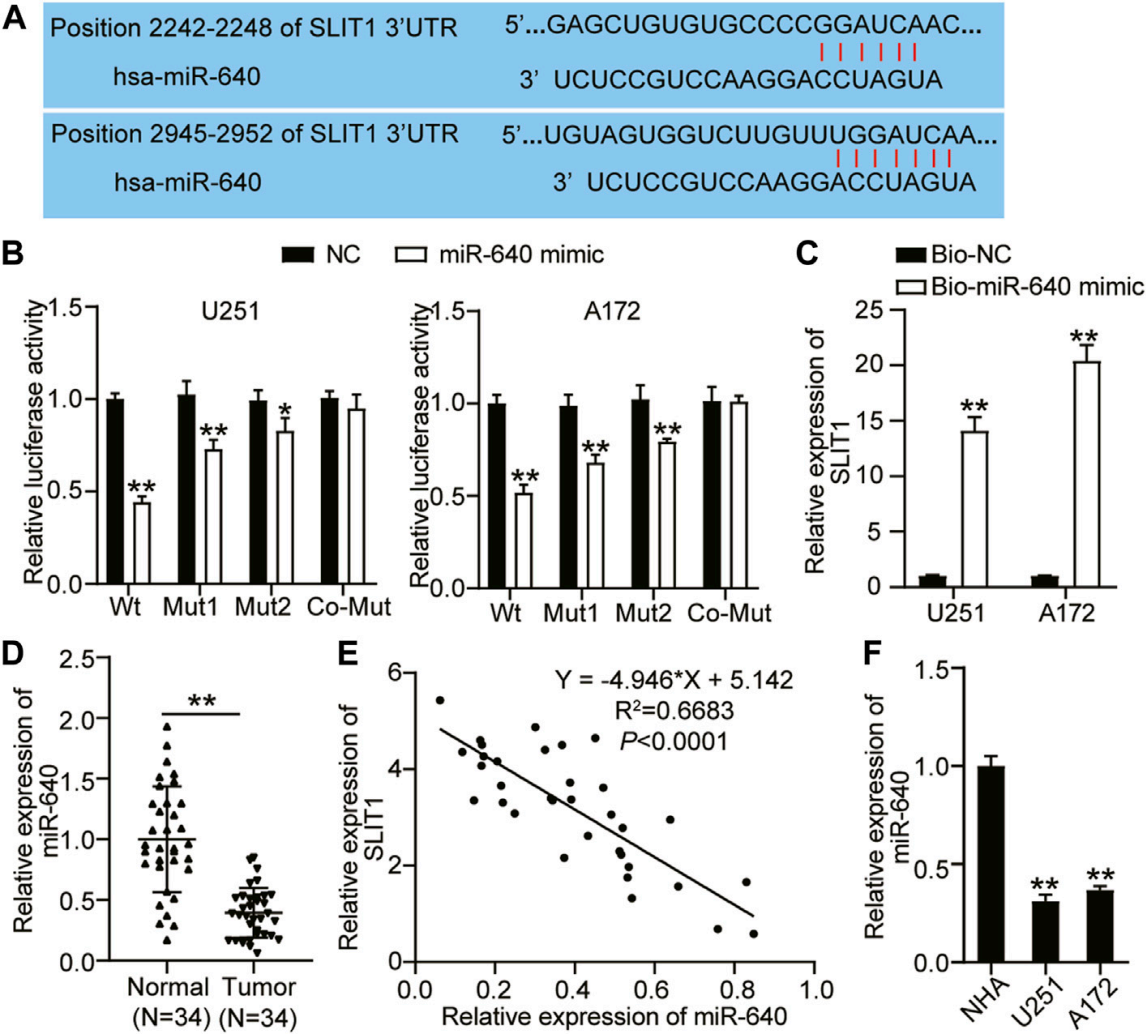
MiR-640 targeted SLIT1. **(A)** The sequence alignment of human miR-640 with the 3′UTR of SLIT1 is shown in TargetScan. **(B)** The luciferase reporter constructs, SLIT1 3′UTR-WT and SLIT1 3′UTR-MUT (Mut1, Mut2 and Co-Mut) were made using the seed sequence of miR-640 matching the 3′UTR of SLIT1 mRNA. U251 and A172 cells were transfected with miR-640 mimic or miR-NC. **(C)** The enrichment of SLIT1 in Bio-miR-640 was measured by RNA pull-down assay in U251 and A172 cells. **(D)** qRT-PCR was carried out to determine the expressions of miR-640 in glioma tissues and matched adjacent normal tissues. **(E)** Pearson correlation analysis of miR-640 and SLIT1 in glioma tissues. **(F)** The expressions of miR-640 in U251 and A172 cells and NHA cell line were evaluated by qRT-PCR. *p < 0.05; **p < 0.001.

miR-640 binds to a specific mRNA and negatively regulates its expression, restricting or promoting tumorigenesis (12). Using TargetScan as a target prediction tool, we identified that SLIT1 and the putative miR-640 contain two binding sites (Figure 3A). We performed a luciferase assay to confirm whether miR-640 can directly interact with SLIT1. We constructed a wild-type SLIT1 luciferase reporter gene with miR-640 binding sites and mutated a single (Mut1 or Mut2) or two (Co-Mut) binding sites of the SLIT1 luciferase reporter gene. U251 and A172 cells were co-transfected with miR-640 mimic or the negative control. Overexpressing miR-640 reduced the luciferase activity of the wild type by about 60% and decreased that of Mut1 and Mut2 by about 30% and 20%, respectively. By contrast, it did not affect the luciferase activity of the Co-Mut relative to that of the control (Figure 3B). Next, we evaluated the miR-640 and SLIT1 binding by performing a biotinylated RNA pulldown assay. The relative expression of SLIT1 mRNA was about 14- and 20-fold higher in the U251 and A172 cell lysates containing biotin-labeled miR-640 than those with only the negative control (Figure 3C). This result indicates that miR-640 binds the 3′ UTR of SLIT1 mRNA. We also quantified the expression of miR-640 and SLIT1 with RT-qPCR to assess how it correlates in glioma tissues. In 34 glioma tissues collected from our patients, the expression of miR-640 decreased in glioma tissues (Figure 3D), and it negatively correlated with SLIT1 expression in the glioma (Figure 3E). Moreover, the expression of miR-640 was lower in the U251 and A172 cells compared with that in the NHA cells (Figure 3F). Therefore, our pull-down experiment and expression analyses suggest that miR-640 binds and negatively regulates SLIT1 expression.

### Loss of miR-640 Activity Reduces Radiosensitivity of Glioma Cells and Stimulates Tumor Progression by Relieving SLIT1 Inhibition

**FIGURE 4:**
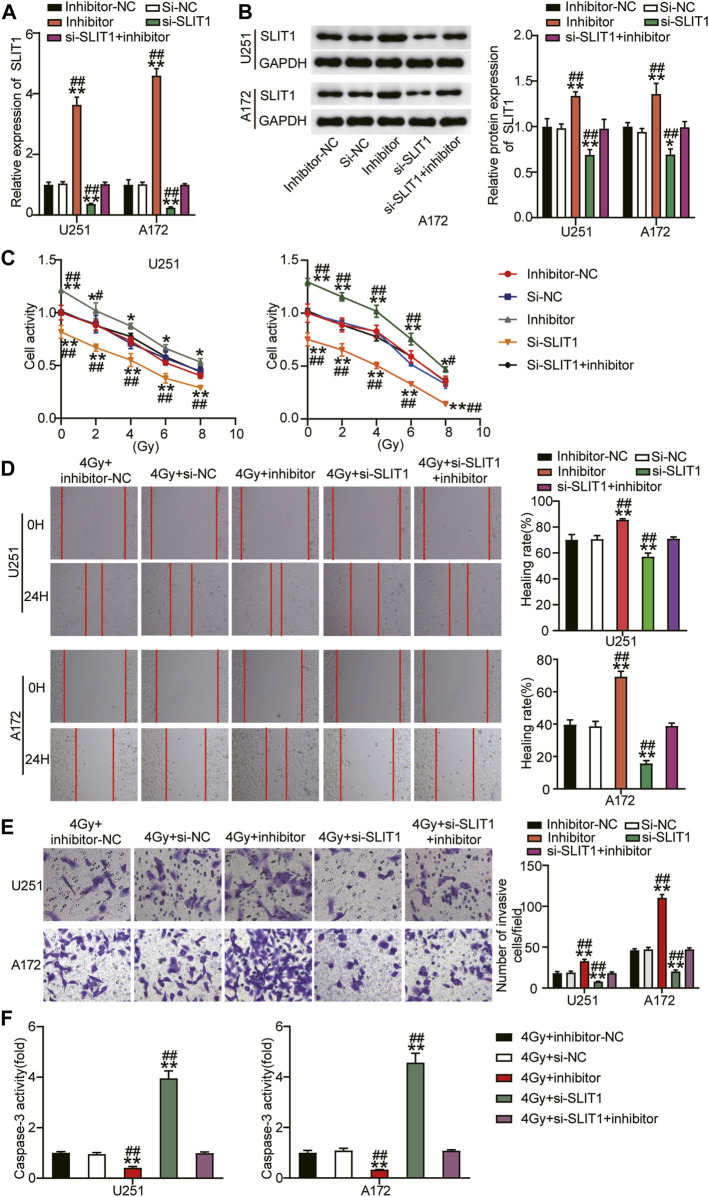
Downregulation of miR-640 enhanced radiosensitivity of glioma cells and inhibited tumor progression by promoting SLIT1 expression. **(A)** The expression of SLIT1 in U251 and A172 cells transfected with miR-640 inhibitor or Si-SLIT1 was determined using qRT-PCR. **(B)** The protein expression of SLIT1 in U251 and A172 cells transfected with miR-640 inhibitor or Si-SLIT1 was determined using Western blot. **(C)** Transfected U251 and A172 cells were exposed to different doses of irradiation, followed by cell viability evaluation by CCK-8 assay. **(D)** U251 and A172 cells with miR-640 inhibitor or Si-SLIT1 transfection were subjected to 4 Gy irradiation, followed by the measurement of cells migration rate by wound healing assay. **(E)** The transfected U251 and A172 cells were treated with irradiation at dose of 4 Gy, followed by the measurement of transwell invasion assay. **(F)** Caspase-3 activity was measured in transfected U251 and A172 cells after 4 Gy irradiation treatment. *p < 0.05, **p < 0.001 vs. 4 Gy + inhibitor-NC/si-NC. ^#^ *p* < 0.05, ^##^ *p* < 0.001 vs. 4 Gy + si-SLIT1+inhibitor.

To further verify that miR-640 alters SLIT1, its expression was assessed by qRT-PCR and Western blot upon transfecting U251 and A172 cells with miR-640 inhibitor. SLIT1 mRNA and protein levels increased after downregulating miR-640 (Figures 4A,B). Conversely, they decreased again after the cells were transfected with si-SLIT1, confirming that miR-640 negatively regulates SLIT1 expression. Moreover, miR-640 inhibitor abolished the inhibitory effect of si-SLIT1 on SLIT1 expression (Figures 4A,B). To explore whether miR-640 and SLIT1 negative regulatory relationship has a practical effect on the radiosensitivity of glioma, we performed a CCK-8 assay and showed that the cell viability of U251 and A172 cells decreased with increasing radiation doses. Upon adding the miR-640 inhibitor, cell viability increased in these cells compared that of the mock-treated cells and reversed the inhibitory effect of si-SLIT1 on cell viability (Figure 4C) across a wide range of doses. Furthermore, when the cells were exposed to 4 Gy radiation, the low expression of miR-640 resulted in increased cell migration rate by about 15% and reversed the si-SLIT1-inhibited cell migration (Figure 4D). The transwell assay also revealed that under low miR-640 expression, the level of cell invasion increased and restored the cell invasion ability inhibited by SLIT1 knockdown (Figure 4E). In addition, the presence of the miR-640 inhibitor significantly decreased caspase-3 activity in U251 and A172 cells under 4 Gy irradiation, whereas it was unaffected after co-transfection with miR-640 inhibitor and si-SLIT1 (Figure 4F). Therefore, we conclude that down-regulating miR-640 reduced radiosensitivity of glioma cells, and relieved the effect of si-SLIT1 on radiosensitivity of glioma cells.

### Micro RNA-640 Inhibition of SLIT1 Enhances Glioma Radiosensitivity by Suppressing Wnt/β-Catenin Signaling Pathway

**FIGURE 5:**
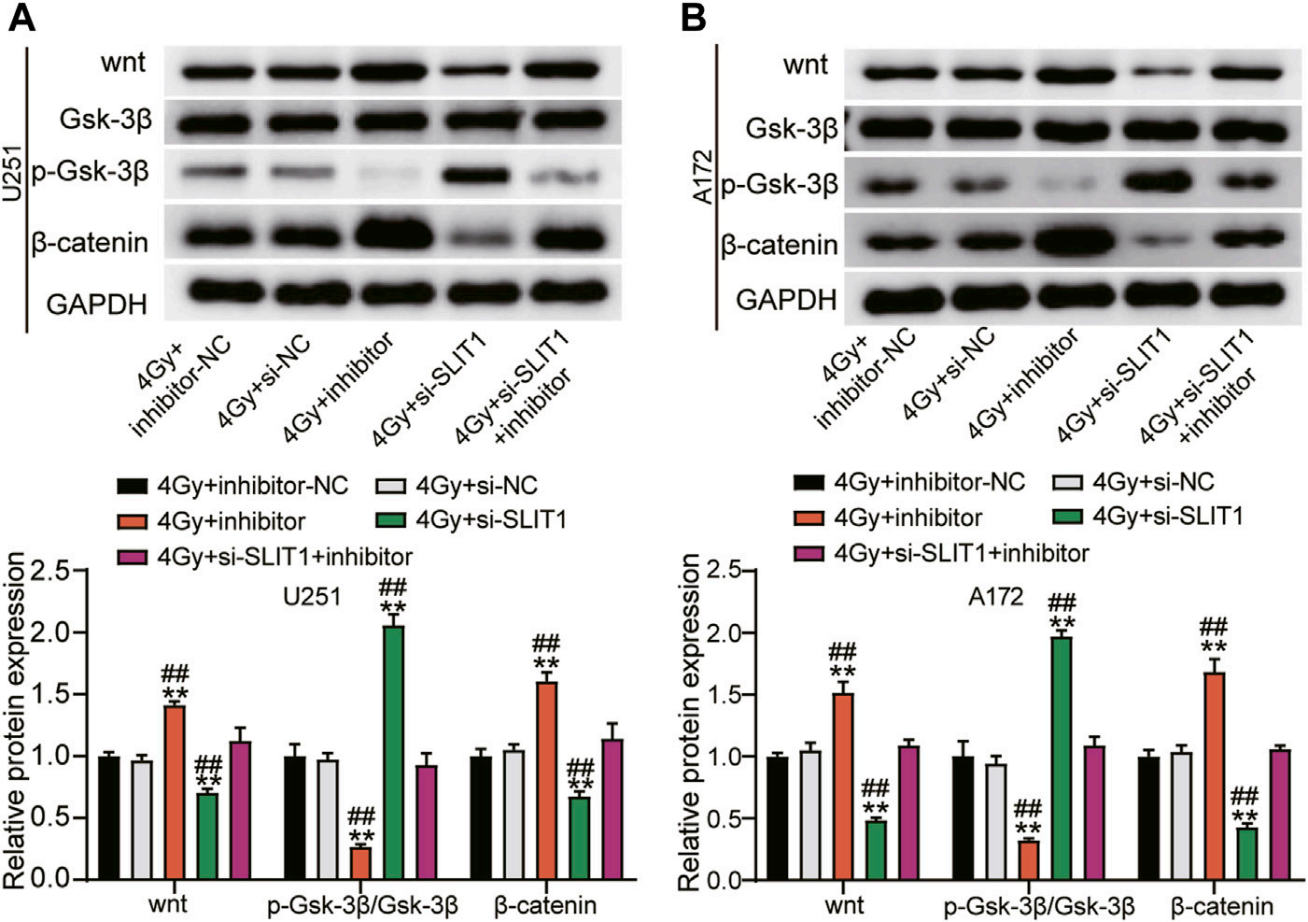
MiR-640 targeting SLIT1 enhanced radiosensitivity in gliomas by inhibiting activation of the Wnt/β-catenin signaling pathway. **(A)** The protein expression of Wnt, β-catenin and p-GSK-3β in U251 cell transfected with miR-640 inhibitor or Si-SLIT1 was determined using Western blot. **(B)** The protein expression of Wnt, β-catenin and p-GSK-3β in A172 cell transfected with miR-640 inhibitor or Si-SLIT1 was determined using Western blot. **p < 0.001 vs. 4 Gy + inhibitor-NC/si-NC. ^##^ *p* < 0.001 vs. 4 Gy + si-SLIT1+inhibitor.

Because the Wnt/β-catenin signaling pathway is usually active in the glioma (22), we investigated whether miR-640 inhibition and SLIT1 silencing affect GSK-3β, Wnt, or β-catenin expression. SLIT1 knockdown in U251 and A172 cells under 4 Gy irradiation significantly decreased the Wnt and β-catenin protein levels but upregulated p-GSK-3β compared with the mock-transfected cells. Conversely, inhibiting miR-640 elevated Wnt and β-catenin levels but decreased p-GSK-3β. Furthermore, it partially reversed the effects of SLIT1 knockdown on Wnt, β-catenin, and p-GSK-3β protein expression (Figures 5A,B). These data imply that miR-640 enhances radiosensitivity of glioma cells by restricting the Wnt/β-catenin signaling via SLIT1 inhibition.

## Discussion

Understanding the adverse effects of radiation tolerance is an important aspect of glioma therapy (7). Therefore, exploring mechanisms of radiation resistance has a high potential for current clinical treatments. Our results suggest that SLIT1 is upregulated in glioma, plays a vital role in promoting glioma development, and is a molecule conferring radiation resistance.

Recent studies have shown that SLIT plays an important role in tumorigenesis, tumor progression, and metastasis (26). Three SLIT encoding genes, SLIT1, SLIT2, and SLIT3, exist in the mammalian genome (27). SLIT1 is located on human chromosome 10q24.1, SLIT2 on 4p15.31, and SLIT3 on 5q34-q35.1 (15). In breast cancer, SLIT2 has a tumor-suppressing effect by enhancing cell adhesion (28). In lung adenocarcinoma, SLIT2 silencing promotes the proliferation and invasion of cancer cells (28). SLIT3 has anticancer effects in thyroid cancer (29), hepatocellular carcinoma (30), and melanoma (31). In this study, we analyzed how SLIT1 affects glioma radiation tolerance. We found that SLIT1 was overexpressed in gliomas, which is inconsistent with the observations reported by Luo et al (32). The discrepancy could have occurred because our glioma tissue samples were radio-treated, while those used by Luo et al. (32) were not. In addition, we observed radiation-induced SLIT1 expression and detected SLIT1 silencing-induced radio-sensitization of the tested glioma cell lines. SLIT1 silencing was also associated with reduced migration and invasion and enhanced apoptosis of the cell lines. These results suggest that low SLIT1 expression inhibits glioma radiation resistance.

Micro RNAs substantially contribute to glioma development (33). MiR-378e, for example, inhibits glioma progression by regulating cell cycle, glycolysis, apoptosis, invasion, and migration (34). MiR-7-5p is downregulated in glioma, inhibiting proliferation, apoptosis, and metastasis (35). Whether miR-640 and SLIT1 are involved in glioma radiation tolerance is unknown. Here, we predicted that miR-640 binds the 3′ UTR of SLIT1 mRNA to block its translation, which we confirmed with the luciferase reporter assay and RNA pulldown assay. SLIT1 expression in glioma tissues and cell lines negatively correlated with miR-640, indicating that miR-640 inhibits SLIT1. Moreover, miR-640-mediated inhibition of SLIT1 promotes the radiosensitivity of glioma cells.

To investigate the underlying mechanism of miR-640 inhibition of SLIT1 in radiation-resistant glioma, we analyzed the Wnt/β-catenin signaling pathway. The Wnt/β-catenin cascade is affected in human cancers, and its overactivation is a marker for glioma (36). Inhibiting the Wnt/β-catenin pathway reduces proliferation and survival in U87 glioma cells (37) while activating it promotes radio-inhibitory sensitivity of glioma cells by increasing the radiotherapeutic survival index (38). However, the mechanism of the Wnt/β-catenin signaling pathway inhibiting radiosensitivity of glioma is poorly understood. Our study revealed that the miR-640 inhibition activated SLIT1, Wnt, and β-catenin but repressed p-Gsk-3β. Previous reports suggest that the mammalian SLIT1 homolog is an evolutionarily conserved target of the Wnt/β-catenin signaling pathway (39). Knocking down SLIT1 caused suppression of the Wnt/β-catenin signaling pathway, indicating that miR-640 restricts the Wnt/β-catenin signaling pathway by targeting SLIT1 to promote glioma radiosensitivity. In conclusion, miR-640 inhibits radio-resistance of glioma, where SLIT1 and Wnt/β-catenin play a central role.

Genes are regulated by multiple miRNAs (40), and one miRNA may regulate various genes (41). Therefore, the miRNA network upstream of SLIT1 and other possible miR-640 targets (e.g., SLIT2 and SLIT3 mRNAs) related to glioma radiation resistance requires further exploration. Moreover, the regulatory pathways upstream of miR-640 in glioma radiation resistance have never been studied and thus should represent our future research efforts.

## Conclusion

In conclusion, miR-640 enhances the radiosensitivity of glioma cells by inhibiting SLIT1 and restraining the Wnt/β-catenin signal pathway. Therefore, the miR-640–SLIT1 axis that regulates Wnt/β-catenin may be a possible therapeutic option for the effective treatment of glioma in combination with radiotherapy.

## Data Availability

The original contributions presented in the study are included in the article/Supplementary Material, further inquiries can be directed to the corresponding author.

## Abbreviations

MicroRNAs (miRNA, miR); HCC, hepatocellular carcinoma; SLIT (SLIT 1, SLIT 2 and SLIT 3); Human glioma cells (U251 and A172); astrocytes (NHA); streptomycin (Gibco); negative control (inhibitor-NC); 75 nM SLIT1 downregulated plasmid (si-SLIT1); negative control (Si-NC); (Bio-miR-640 mimics) and negative control (Bio-NC).
