# Supplementary material for: Micro RNA-640 Targeting SLIT1 Enhances Glioma Radiosensitivity by Restraining the Activation of Wnt/β-Catenin Signaling Pathway
Source: Br J Biomed Sci. 2022 Apr 7;79:10067. doi: 10.3389/bjbs.2022.10067 (PMC9302537; doi:10.3389/bjbs.2022.10067)
Supplement: Supplementary file 1 [file Table1.docx]

Supplementary Table 1. The clinicopathological characteristics of human glioma.

| Characteristics | N (%) |
| --- | --- |
| Age | 16 (47.06%) |
| <40 | 18 (52.94%) |
| ≥40 |  |
| Gender |  |
| Male | 19 (55.88%) |
| Female | 15 (44.12%) |
| WHO Grade |  |
| I | 4 (11.76%) |
| II | 6 (17.65%) |
| III | 13 (38.24%) |
| IV | 11 (32.35%) |
| KPS score# |  |
| <60 | 20 (58.82%) |
| ≥60 | 14 (41.18%) |
| Tumor dimension# |  |
| <6 cm | 22 (64.71%) |
| ≥6 cm | 12 (35.29%) |
| Recurrence or metastasis |  |
| Negative | 9 (26.47%) |
| Positive | 25 (73.53%) |
